# Low-grade appendiceal mucinous neoplasm associated with ulcerative colitis: A case report and literature review

**DOI:** 10.1097/MD.0000000000043955

**Published:** 2025-08-15

**Authors:** Yasuhide Muto, Hitoshi Hara, Seito Shimizu, Tomoki Kido, Ryohei Miyata, Michio Itabashi

**Affiliations:** a Department of Surgery, Social Welfare Organization Saiseikai Imperial Gift Foundation Inc, Saiseikai Kazo Hospital, Kazo, Saitama, Japan.

**Keywords:** case report, colon cancer, laparoscopic colectomy, low-grade appendiceal mucinous neoplasm, ulcerative colitis

## Abstract

**Rationale:**

Colorectal cancer (CRC) development is a critical prognostic factor for patients with ulcerative colitis (UC), making its early detection important. However, early detection of CRC is clinically challenging. This case report describes a patient diagnosed with transverse colon cancer and a low-grade appendiceal mucinous neoplasm (LAMN) in the context of pancolitis-type UC.

**Patient concerns:**

A 59-year-old male patient with a 24-year history of pancolitis-type UC was referred to our hospital after a routine surveillance colonoscopy for the first time in 5 years revealed transverse colon cancer.

**Diagnoses:**

Colonoscopy revealed type 3 advanced carcinoma in the transverse colon and a protruding lesion resembling a laterally spreading granular tumor at the appendiceal orifice. Computed tomography demonstrated enlargement of the appendix.

**Interventions:**

The patient underwent laparoscopic total colectomy, lymph node dissection of transverse colon cancer, ileal pouch–anal anastomosis, and temporary ileostomy.

**Outcomes:**

The patient was diagnosed with stage IIA transverse colon cancer. Additionally, the appendiceal lesion was diagnosed as a LAMN, a relatively novel pathological entity considered a precursor to appendiceal mucinous adenocarcinoma.

**Lessons:**

Although the appendix is part of the colon and can serve as a site for UC-CRC development in patients with pancolitis-type UC, identifying appendiceal lesions through routine surveillance comprising colonoscopy alone may be challenging. In patients with pancolitis-type UC, lesions known as LAMN, which are recognized as precursors of appendiceal mucinous adenocarcinoma, may develop, thereby requiring careful monitoring.

## 1. Introduction

Ulcerative colitis (UC) is the most common form of inflammatory bowel disease (IBD). For patients with UC, especially long-term UC, the risks of colorectal neoplasia and cancer are high.^[1]^ Advanced cancer at the time of the diagnosis of UC often leads to poor prognoses.^[2]^ Early identification and timely management of UC-associated colorectal cancer (UC-CRC) are the most significant clinical procedures associated with the comprehensive care of UC. Surveillance plays an important role in the early detection of UC-CRC, with colonoscopy serving as the recommended screening test; guidelines state the frequency of colonoscopy based on individual risk factors.^[3,4]^ However, colonoscopy, as recommended by current guidelines, may not fully evaluate the appendix, a part of the colon where UC-CRC may occur in patients with pancolitis-type UC. In 2010, the WHO (World Health Organization) classified the majority of appendiceal noninvasive epithelial lesions as low-grade appendiceal mucinous neoplasms (LAMNs).^[5]^ This report describes a LAMN in a patient with UC who underwent laparoscopic total colectomy because of transverse colon cancer development.

## 2. Case report

**Figure 1.**
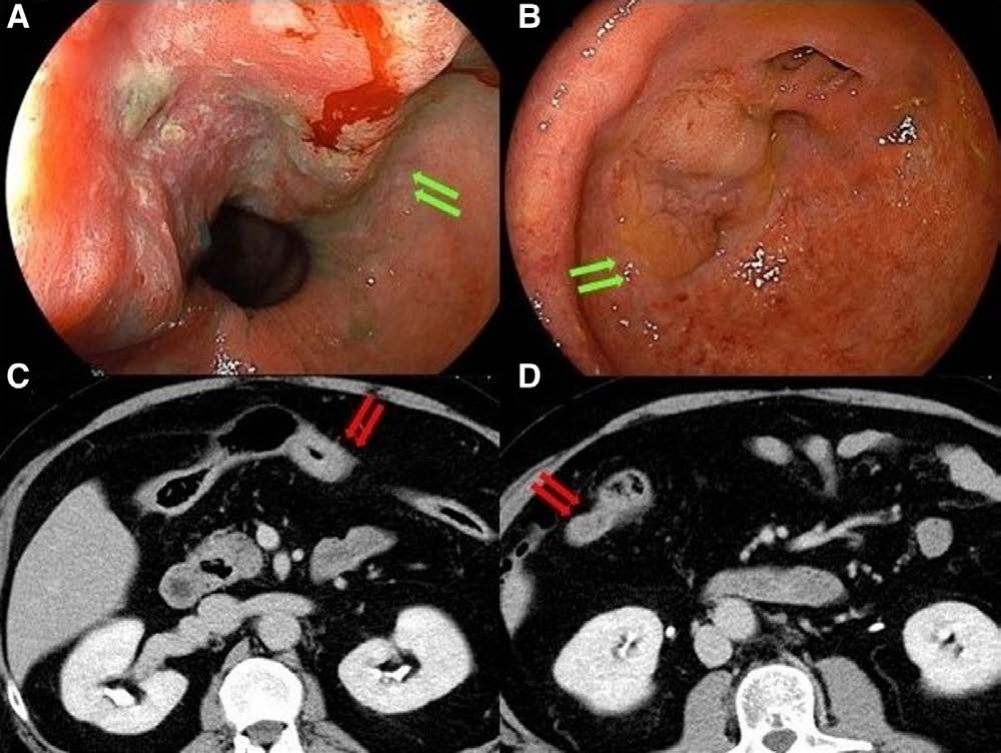
Preoperative examination findings. (A) Colonoscopy findings reveal a type 3 advanced tumor located in the mid-transverse colon (arrow). (B) Colonoscopy findings reveal a LST-like lesion located around the appendiceal orifice in the cecum (arrow). (C) Abdominal contrast-enhanced computed tomography reveals the presence of a tumor within the transverse colon (arrow). (D) Abdominal contrast-enhanced computed tomography findings also reveal an enlarged appendix (arrow). LST = laterally spreading tumor.

**Figure 2.**
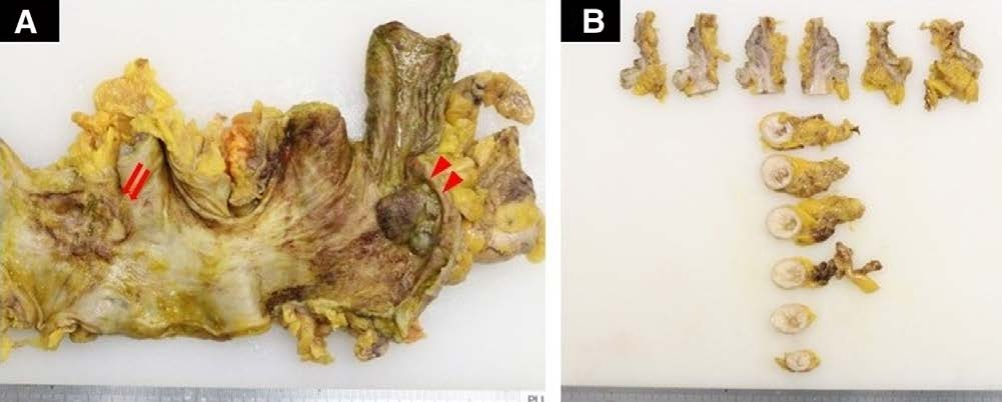
Findings of the resected specimen. (A) A type 5 tumor is identified in the transverse colon (arrow), and the elevated lesion at the appendiceal orifice (arrowhead) detected during preoperative colonoscopy is considered a LST-like lesion. (B) The appendix is swollen, and the cut surface of the appendix reveals the presence of a cystic tumor. LST = laterally spreading tumor.

**Figure 3.**
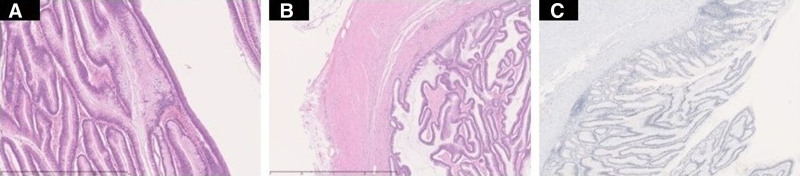
Histopathological findings. (A) The appendiceal tumor is composed of densely packed cells with spindle-shaped nuclei, thus forming villous glandular ducts. (B) Low-grade papillary proliferation and compressive infiltration of mucus-rich cells are observed and the tumor is localized in the appendix, resulting in a diagnosis of a LAMN. (C) The immunohistochemical analysis yields a negative result for p53 staining, which is one of the characteristics of UC-CRC. LAMN = low-grade appendiceal mucinous neoplasm, UC-CRC = ulcerative colitis-associated colorectal cancer.

A 59-year-old man was diagnosed with pancolitis-type UC 24 years before presentation and maintained clinical remission without mesalazine therapy. He used oral prednisolone at a dosage of 10 mg per day because of joint pain. His family history was unremarkable. Surveillance for UC had been discontinued after the hospital where the patient had been monitored closed 5 years earlier. The patient underwent surveillance colonoscopy for the first time in 5 years, resulting in the diagnosis of transverse colon cancer and referral to our department. Colonoscopy revealed type 3 advanced carcinoma in the middle of the transverse colon and a laterally spreading tumor-like lesion at the appendiceal orifice (Fig. 1A and B). Biopsy of the transverse colon lesion confirmed the presence of well-differentiated adenocarcinoma. Contrast-enhanced computed tomography (CT) revealed a tumor in the transverse colon (Fig. 1C) and an enlarged appendix (45 mm × 18 mm) (Fig. 1D) without evidence of lymph node or distant metastases. A serum tumor marker analysis revealed a significantly elevated CA19-9 level (772.2 U/mL) and a normal carcinoembryonic antigen level (5.0 ng/mL). The case was diagnosed as clinical stage IIA transverse colon cancer according to the Union for International Cancer Control 8th edition staging system; additionally, this case was complicated by UC that required total colectomy. Laparoscopic total colectomy, lymph node dissection for transverse colon cancer, ileal pouch–anal anastomosis, and temporary ileostomy were performed. Intraoperative findings included a swollen appendix. The resected specimen (Fig. 2A) revealed a type 5 lesion in the transverse colon (42 mm × 32 mm), an elevated lesion at the appendiceal orifice (31 mm × 22 mm), and an appendiceal cystic tumor (55 mm × 22 mm) (Fig. 2B). A histopathological analysis confirmed that transverse colon cancer was mucinous adenocarcinoma, which is one of the characteristics of UC-CRC (pT3, pN0, M0, stage IIA), and identified the appendiceal lesion as a LAMN (Fig. 3A–C). No lymph node metastases were detected in the ileocecal region. An immunohistochemical analysis revealed that the appendiceal tumor had a negative p53 staining result, which is one of the histological characteristics indicative of UC-CRC. Adjuvant chemotherapy was not performed. The patient underwent successful closure of the ileostomy 3 months postoperatively. Eleven months after surgery, no evidence of colon cancer recurrence was observed.

## 3. Discussion

During UC management, early detection of and intervention for colorectal cancer are the most important clinical procedures because it is a critical factor that influences the patient’s prognosis. UC-CRC accounts for 10% to 15% of all deaths of patients with mortality attributable to IBD.^[6,7]^

The pathogenesis of UC-CRC is distinctly different from that of sporadic CRC. Mir-Madjlessi et al reported that approximately 75% of UC-CRC cases progress through the dysplasia–carcinoma sequence, emphasizing the central role of chronic inflammation-driven molecular alterations in carcinogenesis.^[8]^ Unlike the traditional adenoma–carcinoma pathway observed in sporadic cases, UC-CRC arises in the context of prolonged inflammatory activity and epithelial dysplasia. Dysplasia, a pivotal precursor to UC-CRC development, progresses sequentially; it begins as low-grade dysplasia characterized by mild architectural and cytological abnormalities, advances to high-grade dysplasia marked by pronounced atypia and architectural disorganization, and ultimately evolves to invasive carcinoma. This unique progression highlights the critical need for meticulous surveillance of patients with long-term UC to ensure early detection and timely intervention. Histologically, UC-CRC and sporadic CRC share many similarities, thus making differentiation difficult. A key distinguishing feature of UC-CRC is the higher incidence of poorly differentiated histological subtypes, such as mucinous adenocarcinoma and signet ring cell carcinoma.^[9]^ Immunohistochemistry comprising p53 is a valuable diagnostic tool for UC-associated colon cancer.^[10]^

However, based on histological characteristics alone, distinguishing between CRC caused by UC itself, UC-CRC, and sporadic CRC as a direct cause of somatic mutations and corresponding precancerous lesions is challenging.^[11]^ Despite the strong histopathological similarities of UC-CRC and sporadic CRC, the factors involved in their carcinogenesis differ. The distribution of CRC sites in patients with UC often differs from that of patients with sporadic CRC. UC-CRC tends to occur more frequently in the proximal colon because of extensive colonic inflammation observed with pancolitis, which is a common condition in patients with UC.^[12]^ Surveillance and early detection of CRC in patients with UC are crucial because of their increased risk of cancer over time. Standard colonoscopy is the cornerstone of gastrointestinal tract surveillance and is recommended for the detection of precancerous dysplasia or early malignancies. Studies have demonstrated that colonoscopy enables timely intervention and significantly improves the outcomes of patients with UC-CRC.^[12]^

According to the 2010 WHO classification, LAMNs are mainly noninvasive epithelial lesions that generally present with mucin accumulation in the lumen of the appendix.^[5]^ Additionally, a LAMN is considered a precursor to appendiceal mucinous adenocarcinoma and is characterized by mucin accumulation within the appendix, often without invasion into surrounding tissues. If the LAMN extends into deeper layers, such as the muscularis propria, then active surveillance may be required to detect the potential progression to invasive malignancy. The terms “mucoceles” and “mucinous cystadenomas” are considered obsolete because the term “LAMN” now encompasses them.^[13]^

In 2009, Orta et al reported that 1.56% of patients with IBD had appendiceal cystadenomas; additionally, they noted that appendiceal cystadenomas were especially common in patients with concurrent colorectal dysplasia or cancer.^[14]^ This suggests that IBD with synchronous colorectal dysplasia or cancer is a risk factor for the development of appendiceal cystadenomas, thus implicating these appendiceal tumors as a neoplastic complication of IBD.^[14]^ Many previous appendiceal cystadenomas were included in the LAMN category that was defined in 2010. Therefore, UC can be accompanied by a LAMN, which is a precursor lesion of appendiceal mucinous adenocarcinoma.

Bonomi conducted a systematic review of 34 cases of IBD and AMN in 2024, covering cases from 1997 to 2023, including cases before 2010, when LAMN was first defined by the WHO.^[15]^ Twenty-six (76.5%) patients had UC, with a median age of 52 years and median UC duration of 10 years. Of these, 20 cases of AMN associated with UC were identified as LAMN and 6 cases as adenocarcinoma. In 23 cases with recorded UC subtype and disease activity, 10 had active pancolitis UC. As in this case, 9 cases of LAMN were associated with pancolitis UC, accounting for 34.6% of UC cases. Of the 34 patients, 17 underwent endoscopic examination, and 10 of these had significant endoscopic findings at the appendiceal foramen. This review suggests that AMN may be a neoplastic complication of IBD, and states that in addition to colonoscopy, which is widely used in IBD surveillance, imaging diagnosis such as abdominal ultrasound (AUS) and CT are useful for evaluating appendiceal neoplasms.

Various endoscopic findings at the appendiceal orifice, especially in cases involving appendiceal tumors, are observed at presentation. In some cases, polyps or other neoplastic lesions may be visible at or near the appendiceal opening. However, because of anatomical factors, the diagnosis of appendiceal tumors via lower gastrointestinal endoscopy poses significant challenges. Symptoms such as abdominal discomfort may be present, and lesions can be identified using imaging techniques such as CT or ultrasound. However, these lesions are frequently discovered incidentally during the histopathological examination of resected specimens obtained during acute appendicitis. In this case, total colectomy was performed because of the development of UC complicated by transverse colon cancer, and the histopathological diagnosis confirmed the presence of a LAMN. Because the appendix is part of the large intestine, it can be a potential site of UC-CRC development. In patients with pancolitis-type UC, neoplastic complications of the appendix that are difficult to detect by colonoscopy may be identified through CT or ultrasound examinations.

To the best of our knowledge, since the formal LAMN definition was established in 2010, only 9 cases of LAMNs associated with UC have been reported, including the present case.^[16–22]^ A summary of these cases and ours is presented in Table 1. The sex distribution ratio was 4:5 (male-to-female), the median age of patients was 55 years, and the duration of UC ranged from 12 to 35 years (median, 21 years). Of the 5 cases in which the type of UC was described, 4 cases (cases 3, 4, 8, and our own case) were categorized as the pancolitis type (Table 1). Five patients underwent surgical intervention because of symptoms such as abdominal pain and hematochezia, whereas the remaining 4 patients, including our patient, were asymptomatic. Appendiceal tumors were identified in 4 cases, including ours, during surveillance via lower gastrointestinal endoscopy. A histopathological examination of the surgical specimens confirmed the diagnosis of a LAMN in all cases, but none exhibited the characteristic pathological features of UC-CRC.

**Table 1:** Summary of LAMN cases with UC since the WHO classification in 2010.

No.	Year	Author	Age	Sex	Duration of UC (year)	UC type	Surveillance	Symptoms	Surgical procedure	Pathological findings characteristic of UC-related colon cancer
1	2010	Ghosh et al^[16]^	55	F	NR	NR	Colonoscopy	None	Laparoscopic right hemicolectomy	None
2	2012	Wong and Darwin^[17]^	62	F	NR	NR	Colonoscopy	None	Laparoscopic appendectomy	None
3	2012	Wong and Darwin^[17]^	34	F	NR	pancolitis	NR	Bloody diarrhea	Appendectomy	None
4	2015	Tonolini^[18]^	71	M	NR	pancolitis	NR	Abdominal pain, bloody stool, fever	Laparoscopic appendectomy	None
5	2016	Klag et al^[19]^	63	M	35	NR	Colonoscopy	None	Laparoscopic partial cecum resection	None
6	2020	Davey et al^[20]^	42	M	18	NR	NR	Abdominal pain, bloody stool	Right hemicolectomy	None
7	2022	Abdulghaffar et al^[21]^	48	F	NR	Left-sided colitis	NR	Discomfort on the right iliac fossa	Appendectomy	None
8	2023	Fakheri et al^[22]^	52	F	12	pancolitis	NR	Right lower abdominal pain	Right hemicolectomy	None
9	2025	Our case	59	M	24	pancolitis	Colonoscopy	None	Laparoscopic total colectomy	None

F = female, LAMN = low-grade appendiceal mucinous neoplasm, M = male, NR = not reported, UC = ulcerative colitis, WHO = World Health Organization.

This case report has several limitations. The present LAMN case did not exhibit the histopathological characteristics typically associated with UC-CRC, and the precise relationship between LAMNs and UC remains unclear. Although the review revealed several reports, the possibility of incidental coexistence of UC and LAMN cannot be ruled out. Because a LAMN is a relatively new disease, few reports have described its association with UC. Further case studies and detailed investigations are necessary to elucidate the association between UC-CRC and LAMNs. According to a report by Govaerts et al on appendiceal tumors and pseudomyxoma peritonei,^[23]^ although LAMN is a precursor lesion of adenocarcinoma, most cases follow a slow course, and only approximately 5% of them progress to pseudomyxoma peritonei, which has a significant impact on prognosis. Appendiceal neoplastic complications, such as LAMN, should be carefully monitored during surveillance of the pancolitis-type UC; however, the extent to which they affect prognosis remains unclear.

The appendix is a part of the colon and can serve as a site for UC-CRC development in patients with pancolitis-type UC. In conclusion, appendiceal neoplastic complications – specifically LAMN – which can be difficult to adequately evaluate with colonoscopy alone and are recognized as precursors of appendiceal mucinous carcinoma, may develop in patients with pancolitis-type UC, thereby requiring careful monitoring.

## Acknowledgments

The authors thank Dr A. Kawahara for the supports with the pathological diagnosis and Editage (www.editage.jp) for English language editing.

## Author contributions

**Conceptualization:** Yasuhide Muto, Hitoshi Hara.

**Investigation:** Yasuhide Muto, Hitoshi Hara, Seito Shimizu, Tomoki Kido, Ryohei Miyata.

**Methodology:** Yasuhide Muto, Hitoshi Hara, Tomoki Kido.

**Project administration:** Michio Itabashi.

**Supervision:** Hitoshi Hara, Ryohei Miyata, Michio Itabashi.

**Writing – original draft:** Yasuhide Muto.

**Writing – review & editing:** Hitoshi Hara.
